# Enhancing the Interface Behavior on Polycarbonate/Elastomeric Blends: Morphological, Structural, and Thermal Characterization

**DOI:** 10.3390/polym15071773

**Published:** 2023-04-02

**Authors:** Pedro Veiga Rodrigues, Bruno Ramoa, Ana Rita Torres, Maria Cidália R. Castro, Ana Vera Machado

**Affiliations:** Institute for Polymers and Composites (IPC), Department of Polymer Engineering, University of Minho, 4804-533 Guimarães, Portugal

**Keywords:** polycarbonate, elastomeric phases, interface, toughening

## Abstract

A systematic study was performed to provide better understanding of the effect of elastomeric materials on the behavior of polycarbonate blends (PC). Thus, blends of PC with different amounts of elastomers, such as copolyether ester elastomer (COPE), acrylonitrile–butadiene–styrene (ABS), maleic anhydride-grafted ABS (ABS-*g*-MA), and styrene–ethylene–butylene–styrene (SEBS-*g*-MA) were prepared in a co-rotating twin-screw extruder. The materials were characterized by an electronic microscopy (SEM), an infrared spectroscopy (FTIR), and thermal (DSC) and thermo-mechanical (DMA) techniques. The incorporation of elastomeric phases was observed by changes in the FTIR band’s intensity, whereas a new shoulder of the ester band of COPE at 1728 cm^−1^ indicates the occurrence of a transesterification reaction. Unmodified and modified ABS (5% and 10%) did not affect the glass transition temperature (T_g_) of PC, while 1% SEBS-*g*-MA slightly increased this value. PC/10% COPE showed that a decrease in T_g_ of 25 °C has a result of better compatibilization between both phases, which is visible via SEM. SEM analysis identified three main toughening mechanisms, depending on the type of elastomer. Unlike any other study, this work deepens the knowledge, in a comparative way, to understand the elastomeric effect at the interface and consequently, on the mechanical behavior of PC systems.

## 1. Introduction

Polycarbonate (PC) has been used in several engineering applications, particularly in automotive parts, due to its mechanical properties, as an impact mitigator in energy-absorbing applications, and in others due to its excellent transparency. It presents notch sensitivity and high melt viscosity; thus, one way to overcome these problems is through blending with rubber-containing polymers [1]. This procedure takes place mainly in the melt (melt blending) in a twin-screw extruder with a modular screw that allows for both distributive and intensive mixing of the phases. Over the last two decades, a lot of research has been made on PC blends with different elastomeric materials. A traditional example is blending PC with acrylonitrile–butadiene–styrene terpolymer (ABS), which results in a polymeric system with greater notch sensitivity resistance, heat distortion, and improved processability [2,3]. Melt blending does not exclusively enhance the properties of the blend. It is not uncommon to have a property tradeoff after blending different material systems; for example, improving impact resistance can have a negative effect on the tensile strength. Blending PC with different amounts of ABS has proven to decrease the impact strength of unnotched and notched samples [4]. Nevertheless, these systems have demonstrated to be highly beneficial in preventing the impact failure of samples with sharp notches [2]. Under quasi-static tensile tests, ABS has a depreciation of all tensile parameters [5]. Additionally, PC/ABS blends present a catastrophic failure right after the crack initiation, which is a serious problem for structural applications [6]. Polyester blends with dispersed elastomeric polymers have low miscibility and compatibility due to their chemical nature, which decreases the interaction between both phases and negatively affects the ability to transfer stresses along the matrix. Reactive extrusion has been employed to increase the interaction between incompatible materials and, therefore, improve their mechanical performance. For this, compatibilizers are used to create bridges between each phase. Usually, the polymer backbone has functional groups that will react with the polyester matrix, creating new covalent or hydrogen bonds.

Some compatibilizers are developed by grafting reactive groups, such as maleic anhydride (MA), onto the polymer backbone, which is well described in the literature [7,8,9,10,11]. Usually, the standard procedure is through free-radical grafting, where radicals are initiated with radical generators, such as peroxides. The insertion of MA into the polyolefinic backbone by reactive extrusion has been reported and characterized under different conditions. The number of grafted groups is strongly dependent on several factors, such as the reagent concentration (ratio of peroxide/MA), type of peroxide, processing conditions (temperature, screw speed, residence time, and screw configuration), the presence of co-monomers, and the chemical structure of the polymer [10,12,13]. Regarding polycarbonate blends, polymers with MA groups (synthetized in the laboratory or commercially available) have been used to decrease the surface energy between phases. In the case of PC/ABS blends, compatibilizers with MA grafted on ABS, ethylene–vinyl acetate and SEBS, were successfully used, typically in the range of 1–10 wt%, with huge improvements on the mechanical properties (toughening) [5,14,15,16,17]. The addition of ABS grafted with MA causes a pronounced increase in the impact strength of Izod-notched specimens (an almost seven-fold increase) [18]. Additionally, crack propagation can be delayed after crack initiation, preventing the system from failing catastrophically [6]. The incorporation of small amounts of unmodified and modified styrene–ethylene–butylene–styrene copolymer (SEBS) grafted with MA (SEBS-*g*-MA) shows the benefits on the impact strength and notch sensitivity of PC, with regard to the marginal reduction in the modulus and tensile strength [19,20,21]. The presence of MA groups that react with PC terminal -OH groups provides better stress transfers between the phases.

Additionally, it is possible to take advantage of the reactive extrusion using chemically reactive polymers through the transesterification reaction between ester groups, which improves the interfacial properties. This kind of reactive originates in a copolymer with both parts of each of the incompatible polymer chains. Some studies have reported the importance of this reaction and its effects on miscibility, mechanical, and rheological properties [22,23,24,25]. Contrary to the addition of compatibilizers, this route can provide better phase dispersion and miscibility of the dispersed phase. Sivaraman et al. published a work on blending PC with a thermoplastic copolyether ester elastomer (COPE) through melt blending, where the toughness of these PC blends was greatly increased [26,27]. However, morphology analysis shows the phase separation of COPE, indicating that transesterification did not occur during melt blending. COPE is a commercial grade named Hytrel from DuPont. It is an elastomeric thermoplastic copolymer made of poly(butylene terephthalate) (PBT) and poly(ether glycol) (PEG); therefore, it has block hard and soft segments, respectively [28,29]. Blending COPE with brittle polymers has proven to enhance the blend’s deformation ability through crazing and shear yielding mechanisms [30].

For applications where the materials are subjected to distinct mechanical solicitations, such as automotive parts or protective equipment, the typical uniaxial tensile tests are not sufficient to fully understand the performance of a given part in a specific situation. It is of utmost importance to characterize the materials under different rates of deformation to predict their real behavior. Most of the published articles only disclaim each system alone and do not present a comprehensive comparative discussion of different PC blend systems. To accomplish this, this research performs a systematic blend of PC with elastomeric polymers (ABS, ABS-*g*-MA, COPE, and SEBS-*g*-MA) at small and medium strain rates to better understand the mechanical and fracture mechanisms of these systems. Due to the extensive report, this work is divided into two separate articles, named “Part I” and “Part II”. Part I describes the blending effect on the morphology and structural analysis, glass transition, and dynamic mechanical analysis. Part II details the mechanical evaluation, fracture behavior, and mechanisms, and will be published in the near future.

## 2. Materials and Methods

### 2.1. Materials

Acrylonitrile Butadiene Styrene (ABS) Terluran GP-22 was purchased from BASF (Ludwigshafen, Germany); polycarbonate (PC) Lexan 103R was obtained from Sabic (Riyadh, Saudi Arabia). Styrene–ethylene–butylene–styrene thermoplastic elastomer grafted with maleic anhydride (SEBS-*g*-MA) Taipol 7126 was kindly supplied by TER^®^ AS Chemicals (Hamburg, Germany), and thermoplastic copolyether ester elastomer (COPE) HYTREL 4069 was kindly supplied by DuPont (Wilmington, DE, USA). Table 1 displays some general characteristics of each polymer. Maleic anhydride (MA) with a purity of 99% was acquired from Acros Organics (Geel, Belgium) and the initiator dicumyl peroxide with a purity of 98% (DCP) from Alfa Aesar (Kandel, Germany).

### 2.2. Sample Preparation

#### 2.2.1. Grafting MA onto ABS

ABS was grafted with 2.5 and 5 wt.% of MA using a DCP/MA ratio of 0.2 and named ABS-*g*-MA1 and ABS-*g*-MA2 along the remainder of the manuscript. MA grafting reaction onto ABS was performed in a co-rotating twin-screw extruder (Leistritz, Nürnberg, Germany) using an average barrel melt temperature of 190 °C, a screw speed of 100 rpm, and a throughput of 3 kg/h.

#### 2.2.2. PC Blending

Prior to processing, all polymers were dried overnight in a vacuum oven at 85 °C. Melt blending took place in a co-rotating twin-screw extruder (Leistritz, Germany) at an average melt temperature of 230 °C, 100 rpm, and a throughput of 3 kg/h. The residence time of the material inside the extrusion chamber was assessed by introducing pellets containing a blue pigment. The introduction of the pellets in the feeding zone until visual observation of an intense color change at the die took around 3 min; thus, this value was assumed as the average residence time. After processing, the filament was ground in a knife mill for subsequent processing. Different blend compositions of PC with unmodified and modified ABS, SEBS-*g*-MA, and COPE were prepared as described in Table 2. The blend composition was chosen taking into account the published literature. The amount or range of rubber phase was selected depending on what demonstrated better compromise between toughening and tensile behavior, so it could be tested and compared systematically with the other blends [2,4,18,19,20,26,31,32,33].

#### 2.2.3. Injection Molding

Miniaturized tensile specimens (2 mm thickness, 4 mm gauge width, and 20 mm gauge length) were injected in a Boy 22A injection molding machine (Dr. Boy GmbH & Co. KG, Neustadt-Fernthal, Germany), at a flow rate of 5 cm^3^/s and barrel/mold temperature of 280/80 °C, respectively.

### 2.3. Characterization

#### 2.3.1. Structural Analysis and Grafting Degree

Grafted ABS samples were dissolved in acetone to remove the unreacted MA, precipitated, and washed several times with methanol. Finally, the recovered material was dried at 180 °C for 1 h under constant nitrogen flow. The chemical structure and the grafting degree (GD) were analyzed by Fourier transform infrared (FTIR) spectroscopy (Jasco FT-IR 4100) in transmission mode from a range of 3500–400 cm^−1^. For better representation and visualization of IR spectra, the transmission (%T) signal was converted into absorbance (A) following the formula A = 2 − log_10_(%T). GD (wt.% MA) was quantified by correlating the absorption peaks of the vibrations at 1780 and 2237 cm^−1^ of carbonyl (-C=O) from MA and ABS nitrile groups (-C≡N), respectively. A linear calibration curve (m = 1.35) was built with different ABS and MA compositions to estimate the GD of the purified samples, following Equation (1) [34]:

(1) $$\mathrm{GD}\;\left(wt.\%\mathrm{MA}\right)=m\times \frac{\mathrm{abc}.{\mathrm{CO}}_{1780}}{\mathrm{abs}.{\mathrm{CN}}_{2237}}$$

#### 2.3.2. Fracture Surface and Morphological Characterization

The fracture surface of the tested specimens was studied using an optical microscope DMS 1000 (Leica, Wetzlar, Germany) coupled with a light polarizer. To visually evaluate the specimen stress distribution after injection molding, two light polarizers sheets with perpendicular polarization angles were applied in a light chamber. The morphology of the blends was analyzed by scanning electron microscope (SEM) using a FEI Quanta 400 (FEI, Amsterdam, The Netherlands). Tensile samples were previously fractured in liquid nitrogen and coated with a thin film of gold–palladium.

#### 2.3.3. Thermo-Mechanical Characterization

The storage modulus and dissipation factor of the blends were assessed by dynamic mechanical analysis (DMA) using a DMA TRITON. For this test, the measurements were performed in triplicates, and the gauge length of the tensile specimens was used as a specimen. The distance between grips was 7.5 mm. A load of 1 N was applied at a frequency of 1 Hz in the temperature range of 40 to 180 °C using a heating rate of 2 °C/min.

#### 2.3.4. Thermal Characterization

The thermal behavior was analyzed using a DSC Netzsch 200 Maya (Netzsch, Selb, Germany). The tests were performed under nitrogen atmosphere with a heating rate of 10 °C/min from 40 to 170 °C. Two heating cycles were performed. The first was used to erase the thermal history of the material and the second was used to evaluate the effect of material blending.

## 3. Results and Discussion

### 3.1. Grafting Degree Evaluation and Blend Structural Analysis

**Figure 1:**
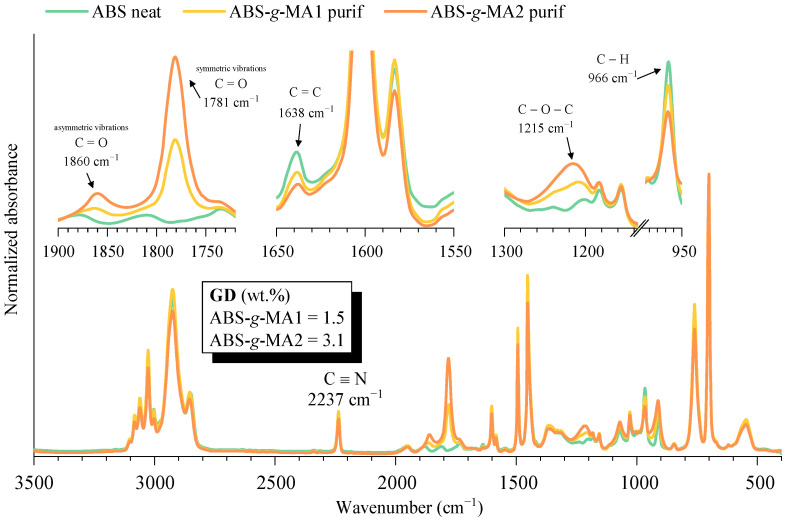
IR spectra of ABS ungrafted and grafted with maleic anhydride.

Figure 1 shows the normalized absorbance IR spectra of the initial and purified ABS grafted with maleic anhydride. Comparing the ABS spectrum with the purified samples, it is possible to detect the appearance of three new absorbance peaks that are related to the successful grafting of MA onto ABS, at 1860 and 1781 cm^−1^ of the C=O asymmetric and symmetric stretching from the cyclic anhydride group, respectively, and 1215 cm^−1^ of the C-O-C vibrations [34,35]. Concerning the polybutadiene (PB) backbone chemical structure, the peak located at 1638 cm^−1^ is assigned to the stretching vibration mode of C=C and the peak at 966 cm^−1^ is assigned to the deformation of vinylic hydrogen [36,37]. Both peak intensities decrease with an increasing GD, which confirms that MA was grafted onto the PB backbone. It is reported that the grafting of MA onto ABS can occur either in the double C=C bond, or by the loss of the vinylic hydrogen of the PB fraction. Therefore, the polyacrylonitrile C≡N bond (2237 cm^−1^) was used to estimate the grafting degree of the modified ABS materials [38]. The calculated grafting degrees were 1.5 and 3.1% for ABS-*g*-MA1 and ABS-*g*-MA2, respectively.

**Figure 2:**
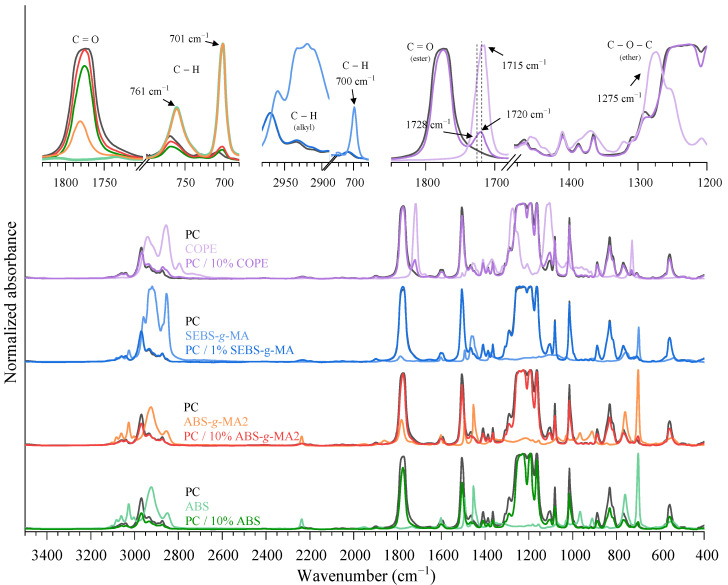
IR spectra of PC blended with 10 wt.% ABS, ABS-*g*-MA2, 1 wt.% SEBS-*g*-MA, and COPE, and their respective base materials.

**Figure 3:**
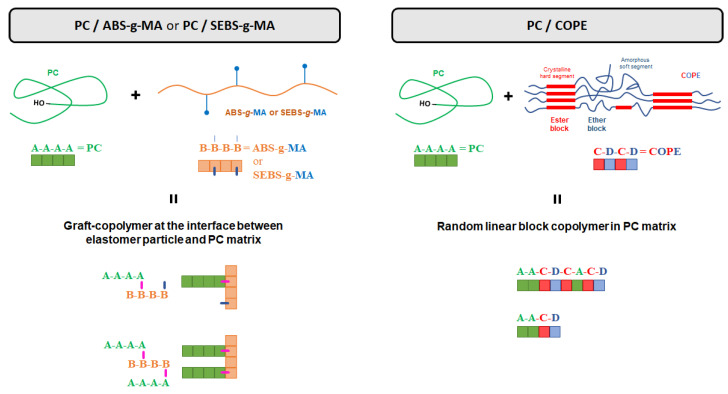
Copolymer structure and dispersion on PC matrix as a result of the reaction between PC/ABS-*g*-MA or PC/SEBS-*g*-MA (**left**) and PC/COPE (**right**).

From the IR spectra of the prepared blends (Figure 2), a decrease in the C=O peak associated with the PC carbonyl groups with the incorporation of 10 wt.% ABS and ABS-*g*-MA, and a shift in the C-H at 761 and 700 cm^−1^ can be observed. A slight increase in the C-H bands in the range of 3000–2800 cm^−1^ and a minor shift at 700 cm^−1^ is the result of the incorporation of 1 wt.% SEBS-*g*-MA. Grafted ABS and SEBS with MA are expected to react with the terminal -OH group of PC, as stated by Balakrishnan et al. [39]. This reaction will not disrupt the core structure of ABS or SEBS (the droplet shape will be maintained), but rather create a chemical connection at the interface of the elastomeric and PC phases (Figure 3).

The ester (1715 cm^−1^) and ether (1275 cm^−1^), characteristic bands of COPE, are detected and correspond to the hard (PBT) and soft (PEG) segments of the copolymer, respectively. Blending with PC promotes a slight deviation of the COPE ester peak to 1720 cm^−1^, and an appearance of a shoulder at 1728 cm^−1^. Around this wavenumber region, it is associated with the vibrational modes of the ester block of COPE. The slight deviation of the COPE ester peak (from 1717 to 1720 cm^−1^) shows an increase in the amorphous form of COPE [40]. However, the shoulder at higher wavenumbers could be an indicator of the formation of a new ester chemical bond. This might be attributed to the occurrence of a transesterification reaction between PC and COPE groups, creating a copolymer of PC-COPE, as shown in Figure 3, which will improve the interface between both polymers [27]. Devaux et al. theorized that the reaction between PC and PBT would show two peaks at 1740 and 1070 cm^−1^ due to the formation of an aromatic ester [41]. The peak at 1290 cm^−1^ of PC is related to the C-O vibrational bonds, which decrease in intensity, and a shift in the C-H vibration around 3000–2800 cm^−1^ and 1500–1300 cm^−1^. This analysis demonstrates that elastomers were successfully incorporated into the PC matrix, and that COPE chemically reacted with PC.

### 3.2. Blends Morphology

It is known that polymer morphology is complex and dependent on several conditions, such as the compatibility between materials, polymer rheology, and processing parameters used during preparation [26,39].

**Figure 4:**
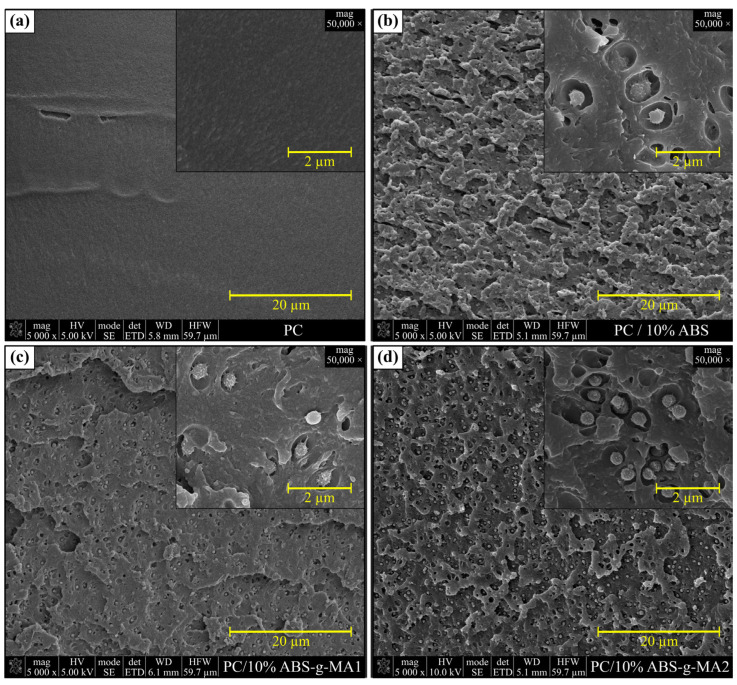
SEM micrographs of cryogenic fractured tensile specimens: (**a**) PC, (**b**) PC/10%ABS, (**c**) PC/10%ABS-*g*-MA1, and (**d**) PC/10% ABS-*g*-MA2 blends.

**Figure 5:**
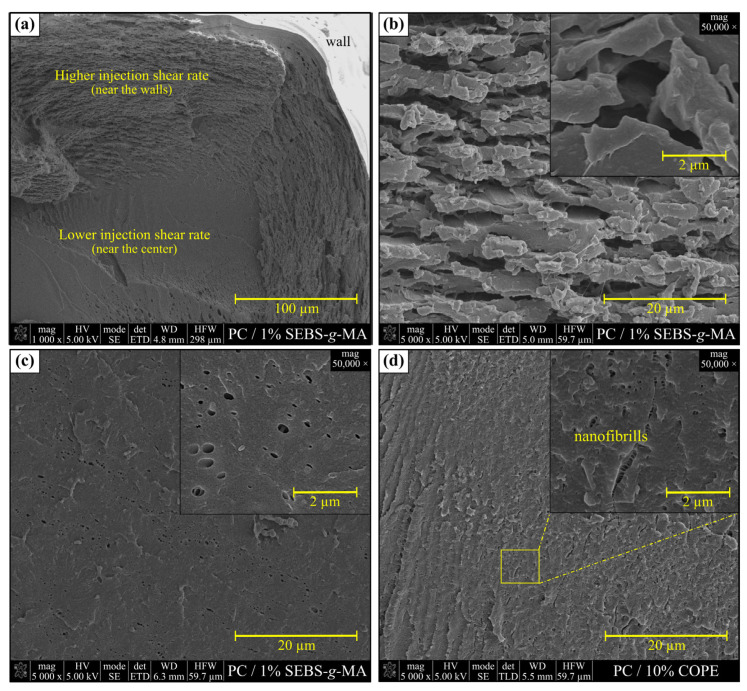
SEM micrographs of cryogenic fractured tensile specimens: (**a**–**c**) PC/SEBS-*g*-MA and (**d**) PC/COPE blends.

The micrographs of the transversal section of PC (a), PC/10%ABS (b), PC/10%ABS-*g*-MA1 (c), and PC/10% ABS-*g*-MA2 (d) are presented in Figure 4, and Figure 5 depicts the PC/SEBS-*g*-MA (a–c), and PC/COPE (d) blends. PC presents a relatively smooth, clean, and featureless surface, where no relevant plastic deformation is visible, indicating the presence of a very brittle fracture under cryogenic conditions. The addition of elastomeric materials has a great impact on the morphology. The ABS phase is dispersed in the PC matrix as droplets, where it can be noticed that a higher grafting degree slightly improves the compatibility when comparing both the non- and modified ABS. While the PC/ABS blend exhibits higher interfacial tension between the two phases, the PC/ABS-*g*-MA micrograph shows a connection between the ABS-*g*-MA droplets and the PC matrix, which might be due to the chemical bond between MA and the -OH end groups of PC [39,42]. Since SEBS-*g*-MA was added in a very low amount (1 wt.%), it was not possible to detect the two phases using SEM. Nonetheless, its influence on the molecular orientation during injection molding on the morphology was noticeable (Figure 5a), where a more lamellar-like structure is observed for higher shear rates near the walls (highly oriented polymer molecules), contrary to the center of the specimen. The noticeable differences between PC and PC/SEBS-*g*-MA micrographs can be attributed to the cavitation phenomenon. In a recent study, it was shown that PC/SEBS-*g*-MA systems would mechanically fail through cavitation phenomena with the appearance of fibrils during deformation [20]. Figure 5b shows visible fibrils that occur mainly under a higher degree of molecular orientation, while rubber cavitation phenomena (formation of voids) are more predominant for a lower degree of molecular orientation, see Figure 5c. The addition of 10 wt.% COPE results in very good dispersion on the PC matrix, since it is not possible to distinguish between the two phases in the micrographs. This helps to corroborate the IR analysis in Figure 2, which claims that the transesterification reaction might have taken place during the blending. This is contrary to Sivaraman et al. results, where PC/COPE systems blended in a single-screw extruder did not promote compatibilization, which could be due to the processing conditions, such as the shear rate, temperature, and mixing intensity, as these are the determinant factors for reactive blending [26]. The crack growth is delayed due to the formation of nanofibrils, which is a result of the stretching of the rubber phase (crazing). Overall, PC/ABS blends show a PC phase with dispersed rubber droplets, while the addition of SEBS-*g*-MA and COPE resulted in a continuous phase, where the cavitation and crazing phenomena were more pronounced. Therefore, a better fracture resistance at negative temperature for PC systems that are reinforced with COPE should be expected.

### 3.3. Thermal Characterization

The values of the analyzed glass transition reported in Table 3 were calculated using the midpoint technique, which is in accordance with the ASTM D3418-15 standard [43]. Commonly, homogeneous polymers only present one glass transition temperature, while heterophase immiscible blends will depict the transitions of each constituent. Thus, this parameter is a good indicator in a polymeric blend [44].

Neat ABS and PC present glass transition temperatures of 106 and 145 °C, respectively. After the modification of ABS with MA, this value appears to decrease. Similar trends of T_g_ have been reported in the literature, where the authors have attributed this to the short flank chain in anhydride maleic in the ABS-*g*-MA, which increases the distance among the polymer chains and increases the free volume [18]. However, no significant difference can be noticed between the two grafted ABS. Thermograms of the blend showed the existence of two T_g_s referring to each blend component (T_g1_ for the ABS and T_g2_ for PC) for the PC/ABS and PC/ABS-*g*-MA blends. In these, T_g1_ has a value higher than that of the neat ABS, and a T_g2_ a value that is slightly lower than the T_g_ of PC, suggesting some compatibility between both polymers (taking into account the error associated with the measurement, ±1). From the previous reference, the authors indicated that the smaller the difference between the T_g_s, the better the compatibility [18]. The T_g_ of PC/SEBS-*g*-MA has a marginally higher value than PC, indicating that a small amount of modified SEBS can constrain the molecular mobility of PC chains. The addition of COPE into the PC matrix substantially decreases the value of T_g_, suggesting good compatibility between both polymers.

### 3.4. Thermo-Mechanical Analysis

**Figure 6:**
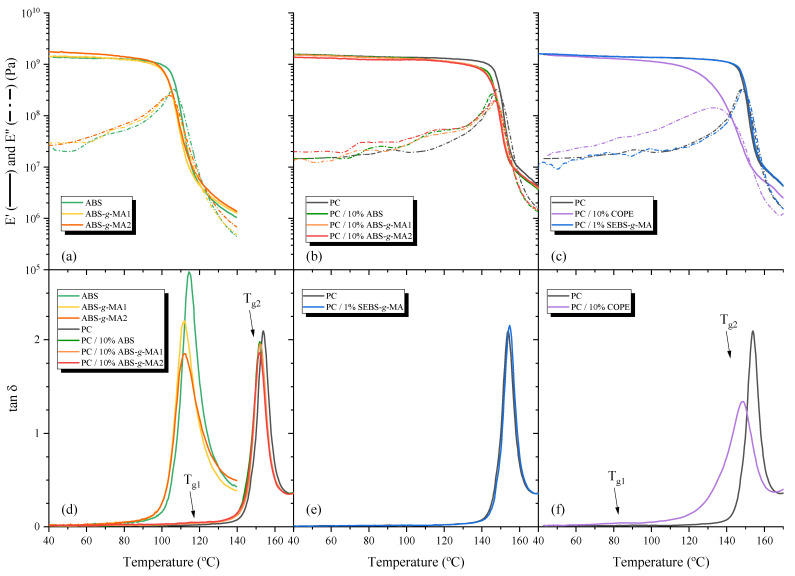
Dynamic mechanical analysis of (**a**,**d**) non and modified ABS, PC blends with ABS and ABS-*g*-MA (**b**,**d**), SEBS-*g*-MA and COPE (**c**,**e**), and PC/10% COPE deconvolution curves (**f**).

The thermo-mechanical characterization of the neat polymers and polymer blends is presented in Figure 6, and the DMA T_g_ values are presented in Table 3.

The effect of grafting MA onto ABS can be noticed in Figure 6a, where the ABS storage modulus ($E^{\prime }$) is relatively constant up to 90 °C. After, a sharp drop is observed as the temperature increases, and tan δ peak appears at a temperature of around 114 °C (Figure 6d). This peak is shifted to a lower temperature (111 °C) for the ABS-*g*-MA samples due to MA grafting. The damping factor area decreases as the GD increases, which is expected because the elastomeric phase of ABS loses part of its ability to dissipate the energy after grafting [45]. The mobility of the elastomeric phase becomes more restricted, causing an increase in the storage modulus *E*’. The T_g_ values obtained through DMA are in agreement with the DSC data, the presence of grafted MA might increase the distance between the polymer chains, thus causing a decrease in T_g_.

The temperature dependence of $E^{\prime }$ on PC depicted in Figure 6b presents a small decrease up to a temperature of 120 °C, after which an inflection can be noticed followed by a non-linear decrease in the modulus up to 170 °C. The tan δ peak (154 °C) associated with a broad α-relaxation can be detected starting at 120 °C and ending at 165 °C (Figure 6e). PC blends with unmodified and modified ABS exhibited a small decrease in the storage modulus at around 110 °C due to the presence of the ABS phase having a noticeable reduction in $E^{\prime }$ with the increasing temperatures before reaching the α-transition of PC. The T_g_ value of the ABS component is not well identified due to its low percentage in the blend, but its influence can be noticed by the increase in E″ (Figure 6b) between 100 and 130 °C, which is due to the chain mobility of the SAN fraction of ABS [46,47]. A small shift in the maxima of the PC α-transition to smaller temperatures is perceived, which is more noticeable for blends with modified ABS (Figure 6d), which confirms the increase in compatibility between both polymers.

The effect of SEBS-*g*-MA on the dynamic mechanical properties of PC can be found in Figure 6c,e, and both the storage modulus and tan δ have remarkably similar behavior, with a small shift to higher temperatures and an increase in the magnitude of the tan δ peak. The same increase in T_g_ was observed in DSC and DMA, suggesting that the small rubber particles of SEBS-*g*-MA constrict the molecular movement of PC chains.

The addition of 10 wt.% COPE produces a substantial difference in the elastic modulus. The value of $E^{\prime }$ starts to decrease at 60 °C, after which the inflection can be detected with a non-linear decremental behavior up to 170 °C. The damping factor of the blend with COPE can be seen to start at ca. 70 °C and finish at ca 160 °C. Two tan δ peaks are detected at 84 °C and 148 °C. The first peak could be attributed to the PEG block of COPE, while the second peak is related to the new polymer structure formed between PC and the PBT block of COPE. Additionally, the peak of the PC phase (154 °C) is not detected. The tan δ curve of PC/10%COPE has a lower damping intensity with a much broader peak, which is in agreement with results discussed above.

DMA sensitivity is greater than DSC for thermal transition detection, since these types of transitions are more noticeable in the mechanical properties rather than in the thermal capacity, which explains the differences between the PC/COPE T_g_ values determined with DMA (148.6 °C) and DSC (138.9 °C) [48].

## 4. Conclusions

The results obtained in this systematic study of blends of PC and ABS, ABS-*g*-MA, SEBS-*g*-MA, and COPE confirmed the conclusion that in all the cases, a chemical reaction took place at the interface. However, it was different for the polymers grafted with MA and COPE, which could be noticed in the morphology. DSC and DMA data also suggest a higher level of compatibility in PC/COPE than the others. These results will be of great importance to the discussion in Part II of this work, since the mechanical behavior of polymer blends highly depends on the interfacial tension between the elastomeric phase and the PC matrix. Moreover, these findings overcome some of PCs drawbacks.

## Figures and Tables

**Table 1:** Polymer characteristics according to datasheet.

Polymers	ρρ (g/cm^3^)	MVR (cm^3^/10 min)	MA (%)
PC (Lexan 103R)	1.20	6 (300 °C/1.2 kg)	-
ABS (Terluran GP-22)	1.04	19 (220 °C/10 kg)	-
SEBS-*g*-MA (Taipol 7126)	n.a.	15–25 g/10 min (230 °C/5 kg)	1.2–1.8
COPE (Hytrel 4069)	1.11	n.a.	-

n.a.—not available.

**Table 2:** Blend composition ratios.

(wt.%/wt.%)	PC	ABS	ABS-*g*-MA1	ABS-*g*-MA2	SEBS-*g*-MA	COPE
PC/5% ABS PC/10% ABS	95	5				
	90	10				
PC/5% ABS-*g*-MA1 PC/10% ABS-*g*-MA1	95		5			
	90		10			
PC/5% ABS-*g*-MA2 PC/10% ABS-*g*-MA2	95			5		
	90			10		
PC/1% SEBS-*g*-MA	99				1	
PC/10% COPE	90					10

**Table 3:** Thermal properties of initial polymers and blends, using DSC and DMA.

Sample *	DSC		DMA	
	T_g1_ (°C)	T_g2_ (°C)	T_g1_ (°C)	T_g2_ (°C)
	Mid-Point	Mid-Point	Peak	Peak
ABS	106	-	114	-
ABS-*g*-MA1	104	-	112	-
ABS-*g*-MA2	104	-	112	-
PC	-	145	-	154
PC/10% ABS	112	145	116	152
PC/10% ABS-*g*-MA1	111	144	117	152
PC/10% ABS-*g*-MA2	112	144	118	152
PC/1% SEBS-*g*-MA	-	147	-	155
PC/10% COPE	-	139	84	149

* Tg values were calculated as follows: mid-point for DSC, and tan δ peak for DMA.

## Data Availability

The data presented in this study are available in the article.
